# Patient Portal Use Near the End-of-Life

**DOI:** 10.1093/geroni/igab046.2857

**Published:** 2021-12-17

**Authors:** Jennifer Portz, John David Powers, David Bekelman, Megan Baldwin, Alejandra Casillas, Jean Kutner, Elizabeth Bayliss

**Affiliations:** 1 University of Colorado, Aurora, Colorado, United States; 2 Kaiser Permanente Colorado, Kaiser Permanente Colorado, Colorado, United States; 3 Kaiser Permanente Colorado, Denver, Colorado, United States; 4 UCLA David Geffen School of Medicine, Los Angeles, California, United States

## Abstract

Use of patient portals, personal health information websites linked to electronic health records, in seriously ill populations is unknown, as is use by caregivers. We described portal use patterns among adults with serious illness nearing end-of-life and their caregivers within Kaiser Permanente Colorado. Inclusion criteria were: 1) seriously ill patients (defined by KP’s “Care Group”), □18 years of age, who were registered for the portal, and died between 1/1/2016-6/30/2019; and 2) caregivers of these patients, □18 years of age, registered for a proxy account. Data included user characteristics and portal use metrics summarized monthly over the 12-month period prior to death. Models included an unadjusted linear trend of the days used by month using a generalized estimating equation Poisson model with a log link and an autoregressive correlation structure of order 1. We identified 6,517 seriously ill patients with portal registrations; 163 of these patients had proxy caregivers. Patient users were 77 years old, mostly frail and White, and caregivers were predominantly female. Average days of use among patients was 42.4 days and <1 day among their caregivers. Number of days used significantly increased by 0.7% per month from twelve months to one month prior to death (95% CI: 0.4%-1.0%; p-value <.0001) and peaked 3 months prior to the patient’s death. Average use was high in comparison to previous portal research and suggests that as the patient approaches death portal use increases. Future research should explore how portals may serve as indicators for identifying and addressing end-of-life care needs.

